# Items

**Published:** 1891-04

**Authors:** 


### Items.

On a recent occasion George Bancroft, the historian, told a bevy of young girls the secret of long life lay in never losing one's temper. If you will never get angry, he said, you will live to be ninety.

Washing the face and hands with pure water aided with a wash rag or sponge, and drying thoroughly with a very soft towel, is, perhaps the best way to keep a good skin.

Water that is clear is not always pure. It may contain the seed germs of disease.

We have seen men and women increase their lung power by five minutes exercise night and morning. Stand straight up on the balls of the feet, head thrown back, and inhale deeply, first inflating the lower part of the lungs then the upper. Then expire slowly, letting the chest sink first and the lungs. Do this fifteen times, morning and evening, and you will be well repaid for the time spent, by fewer colds and little catarrh.

